# Remitting seronegative symmetrical synovitis with pitting edema syndrome with two different underlying malignancies: A case report

**DOI:** 10.1097/MD.0000000000042091

**Published:** 2025-04-04

**Authors:** Samar Alharbi

**Affiliations:** a Department of Medicine, College of Medicine, Taibah University, King Salman Medical City, Medina, Saudi Arabia.

**Keywords:** arthritis, corticosteroids, malignancy, pitting edema, RS3PE, seronegative

## Abstract

**Rationale:**

Remitting seronegative symmetric synovitis with pitting edema is a rare, benign disorder symmetrically affecting the peripheral joints. It begins with acute-onset pitting edema on the dorsum of the hands and feet, and primarily affects elderly men and has a favorable response to low-dose steroids. It may also be a paraneoplastic symptom of an underlying hidden cancer; hence, thorough clinical evaluation is required.

**Patient concerns:**

A 58-year-old woman who presented with acute-onset polyarthritis and pitting edema affecting both hands and feet. Based on clinical and laboratory findings, remitting seronegative symmetric synovitis with pitting edema was diagnosed. A thorough clinical investigation led to a diagnosis of papillary thyroid carcinoma and adenocarcinoma of unknown origin.

**Diagnoses:**

Based on clinical and laboratory findings, remitting seronegative symmetric synovitis with pitting edema was diagnosed. A thorough clinical investigation led to a diagnosis of papillary thyroid carcinoma and adenocarcinoma of unknown origin.

**Interventions:**

The patient initially received prednisolone; when she faced difficulty in maintaining remission at a dose of <12.5 mg, hydroxychloroquine was added. Subsequently, the corticosteroid dose was tapered successfully without further arthritis flares. She also received palliative chemotherapy for an unknown primary.

**Outcomes:**

Five rounds of chemotherapy successfully reduced the size of bilateral inguinal lymphadenopathy and garnered resolution of the small bilateral external iliac lymph nodes. Both corticosteroid and hydroxychloroquine discontinued. The patient remained stable and cancer-free at the 9-month follow-up.

**Lessons:**

Physicians should be knowledgeable about the distinct signs and symptoms of remitting seronegative symmetric synovitis with pitting edema and investigate patients suspected of having the syndrome for hidden cancers.

## 1. Introduction

Remitting seronegative symmetrical synovitis with pitting edema (RS3PE) is rare inflammatory arthritis typically found in the elderly population; it was first described in 1985 by McCarty et al.^[1]^ The syndrome is characterized by the acute-onset of symmetrical polyarthritis involving the hands and/or feet, with associated pitting edema and negative rheumatoid factor (RF).^[2]^ It typically affects elderly individuals aged over 60 years with a male-to-female ratio of approximately 2:1.^[3]^ The global incidence of RS3PE is unknown and standardized diagnostic criteria are yet to be established. Because of its diverse clinical manifestations and possible triggers, RS3PE is categorized into 3 subtypes: idiopathic, drug-induced, and infection-associated. Case reports and reviews on RS3PE outline a range of clinical features and probable associations, such as different types of solid-organ tumors and hematological malignancies, as possible paraneoplastic syndromes. Rheumatic diseases such as polymyalgia rheumatica, late-onset rheumatoid arthritis, Sjögren’s syndrome, and temporal arteritis have also been reported to be likely associated.^[4]^ Here, I discuss a case of a patient diagnosed with RS3PE who presented with symmetric polyarthritis and pitting edema, responded quickly to corticosteroids, and was found to have 2 different underlying malignancies.

## 2. Consent for publication

The patient provided a written signed consent for the purpose of publication. Ethical approval was waived by the local Ethics Committee.

## 3. Case presentation

The patient was a 58-year-old Saudi woman with over 10-years of being treated for type 2 diabetes mellitus. Her last glycosylated hemoglobin (HbA1C) level was 6.5. The patient had no psychological or family history of autoimmune or rheumatological disease. She presented to our rheumatology clinic in March 2023 with painful swelling in both hands and feet, which had started suddenly 2 weeks ago. Before her visit to our clinic, she had been admitted to our hospital because of diarrhea, abdominal pain, and constitutional symptoms and treated with antibiotics. However, her condition did not improve. She reported bilateral pain in her wrists, metacarpophalangeal and proximal interphalangeal joints, and ankles accompanied by swelling, which affected the dorsum of both hands and feet. In addition, she had considerable early morning stiffness that lasted for several hours and affected her functioning. There were no associated features of connective tissue disease. She had ongoing diarrhea, low-grade fever on and off, and fatigue and had lost 8 kg of weight in the previous 2 months.

The patient was in a wheelchair, unable to move, and looked unwell; however, she was afebrile and vital signs were stable. Further, she had active synovitis in her wrists, metacarpophalangeal, proximal interphalangeal, metatarsophalangeal joints, and ankles bilaterally. She also had pitting edema on the dorsum of her hands and feet. Physical examination revealed bilateral inguinal lymph node enlargement, which was greater on the left side than on the right. No cervical or axillary lymphadenopathy or hepatospenomegaly was observed. Other systemic examination results were unremarkable.

**Figure 1.**
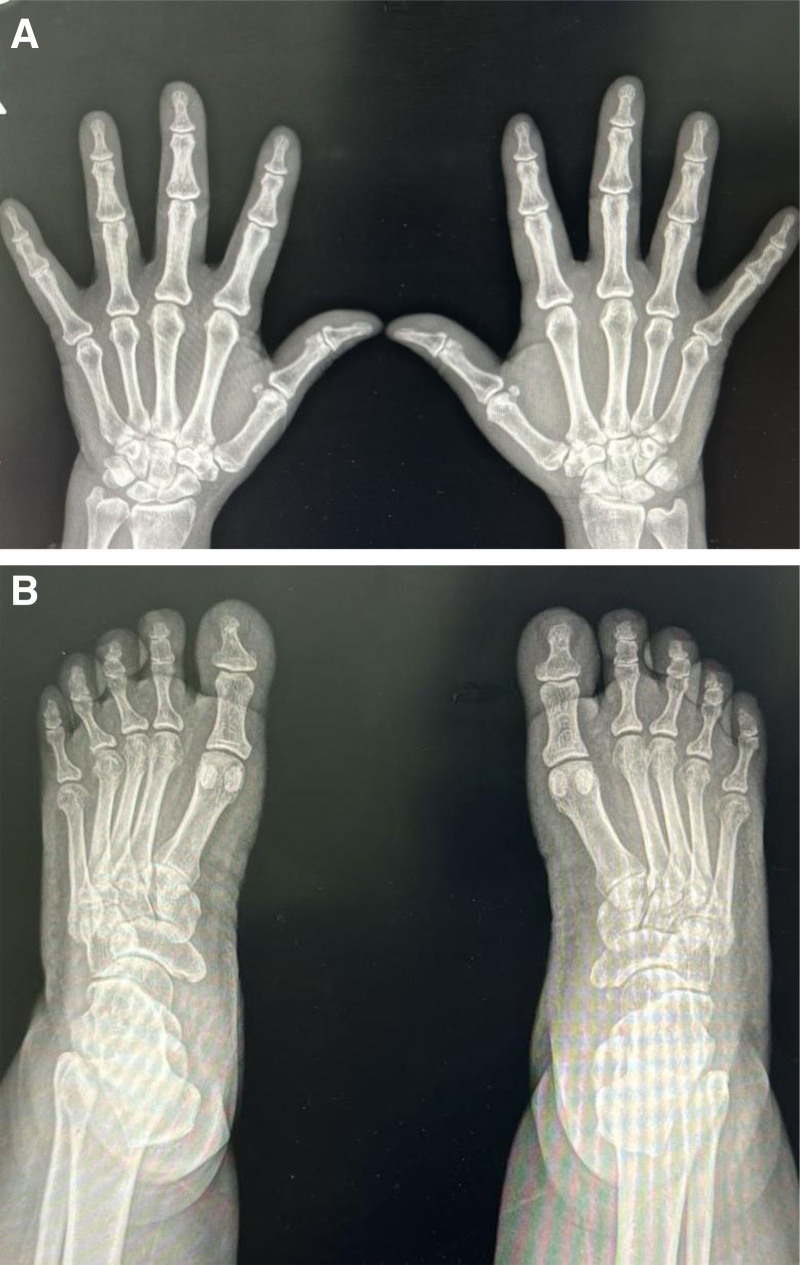
Hand and foot radiographs.

Initial laboratory test results revealed normal white blood cell and platelet counts, a hemoglobin level of 9 g/dL (12–15 g/dL) with normochromic and normocytic anemia, an erythrocyte sedimentation rate of 120 mm/h (normal range: 0–25 mm/h), and C-reactive protein levels of 136 mg/L (normal range: 0–5 mg/L). Renal and liver function test results were normal and so were creatine kinase levels and peripheral blood smear test results. Moreover, blood, urine, and stool cultures and tuberculin skin test results were negative. The patient also tested negative for infection with cytomegalovirus, Epstein–Barr virus, human immunodeficiency virus, and *Salmonella* (Widal test) and *Brucella* species; hepatitis B and C; and malaria. A rheumatology work-up, including antinuclear, RF, anti-cyclic citrullinated peptide, antineutrophil cytoplasmic antibodies, was negative; extractable nuclear antigen panel tests were also negative. HLA B27 was also cheeked and tested negative. Computed tomography (CT) of the kidneys, ureters, and bladder showed bilateral inguinal masses more on the left side, suggesting lymph nodes, the largest of which measured 2.5 × 2 cm, which prompted clinical correlation and further evaluation. Colonoscopy and biopsy findings were unremarkable. Radiographs of hand (Fig. 1A) and foot (Fig. 1B) joints showed soft-tissue swelling without erosive changes.

The patient was admitted to our hospital for further investigation and started on prednisolone 20 mg once daily. Two days later, the joint symptoms and pitting edema improved markedly, and she was able to ambulate. Based on her acute presentation, quick response to corticosteroids, and initial laboratory test results, which included high levels of inflammatory markers and negative RF, RS3PE was highly suspected. Hence, inguinal lymph node biopsy was performed, since bone scan and mammogram results were negative. Thyroid ultrasonography revealed a right thyroid nodule. Fine needle aspiration/excisional biopsy of the thyroid revealed papillary thyroid cancer. Inguinal lymph node histopathology was consistent with adenocarcinoma of unknown primary origin, and immunohistochemistry favored cancer of the breast or gynecological origin (also that of lung and pancreaticobiliary origin). Positron emission tomography findings were unremarkable.

The steroid dose was gradually tapered; at a dose of 12.5 mg, she developed active synovitis in both hands (more in the left than in the right) and both feet along with mild pitting edema. The patient was started on hydroxychloroquine and her prednisolone dose was increased back to 20 mg. Magnetic resonance imaging of the left hand showed evidence of effusion in the proximal interphalangeal joint of the left index finger. The patient underwent total thyroidectomy without complications.

Subsequently, the prednisolone dose was tapered successfully (2.5 mg weekly) until a dose of 5 mg was reached; the daily hydroxychloroquine dose was 200 mg. The patient was observed and assessed multiple times by the oncology team. Contrast-enhanced CT showed an interval increase in the size of the bilateral inguinal lymph nodes and stable, small external iliac lymph nodes. There was no evidence of a primary neoplastic process or metastasis elsewhere in the abdomen and pelvis. The patient was started on palliative chemotherapy for an unknown primary tumor. Restaging with CT of the chest, abdomen, and pelvis after the fourth cycle showed an interval decrease in the size of the previously described bilateral inguinal lymphadenopathy and resolution of the small bilateral external iliac lymph nodes. The patient completed 5 cycles of chemotherapy. Prednisolone was tapered gradually and stopped. One month later, hydroxychloroquine treatment was also discontinued.

In summary, the patient initially received prednisolone; when she faced difficulty in maintaining remission at a dose of <12.5 mg, hydroxychloroquine was added. Subsequently, the corticosteroid dose was tapered successfully without further arthritis flares. After 12 months of using this combination, she was able to discontinue both treatments. The patient was seen 9 months later and was doing well without any joint symptoms. Furthermore, she was undergoing regular oncology follow-up and was stable.

## 4. Discussion and conclusions

RS3PE is a rare type of inflammatory arthritis that predominantly affects men. It was initially described by McCarty et al in 1985 as a separate subtype of late-onset seronegative rheumatoid arthritis. Dr McCarty initially referred to the condition as “boxing glove hand” because of the substantial swelling observed.^[1]^ Ten patients (8 elderly men and 2 elderly women) reported symmetrical acute-onset polysynovitis accompanied by edematous dorsa of the hands.^[1]^ All patients tested negative for RF, and no bone erosion was observed on imaging.^[1]^ The prevalence of RS3PE is unknown; however, a study conducted in Japan reported an incidence of 0.09% in 3347 individuals.^[5]^

Diagnosing RS3PE is a process of excluding other disorders rather than having a definite test. Nevertheless, Olive et al suggested the following diagnostic criteria for this syndrome: bilateral pitting edema in both hands, sudden development of polyarthritis, age >50 years, and seronegative RF.^[6]^ In this case, a 58-year-old woman who presented with acute-onset polyarthritis, pitting edema affecting both hands and feet, a high erythrocyte sedimentation rate and C-reactive protein level, and nonerosive changes on radiography. She responded quickly to corticosteroid treatment. All previous findings in her case supported the diagnosis of RS3PE.

Although the exact pathophysiology of RS3PE is unknown, vascular endothelial growth factor is thought to play a role by increasing vascular permeability, which causes polysynovitis and pitting edema in the extremities.^[7]^ Patients with RS3PE have higher vascular endothelial growth factor levels than those with rheumatoid arthritis or other connective tissue illnesses.^[7]^ The HLA-B7 haplotype has been linked to the syndrome in 59% of cases, whereas the HLA-A2 haplotype has been linked in 76% of cases.^[8]^

Owing to the wide range of possible causes and clinical manifestations, RS3PE has been divided into 3 subtypes: drug-induced, infection-associated, and idiopathic. The drug-induced form is mainly associated with the use of dipeptidyl peptidase-4 inhibitors, insulin, and rifampicin.^[9]^ The type caused by infection appears to be linked to either parvovirus or *Streptobacillus moniliformis* infection.^[9]^

RS3PE can mimic multiple conditions such as polymyalgia rheumatica, elderly onset rheumatoid arthritis, systemic lupus erythematosus, systemic sclerosis, and mixed connective tissue disease.^[8]^ It can also evolve into other connective tissue diseases such as seronegative rheumatoid arthritis, Sjogren syndrome, and relapsing polychondritis.

In addition to RS3PE, we also took reactive arthritis and seronegative rheumatoid arthritis into consideration as differential diagnoses in this case because of the similarities between the 3 conditions. It is crucial to differentiate between reactive arthritis, seronegative rheumatoid arthritis, and RS3PE due to their similarities. RS3PE is characterized by a rapid response to corticosteroids, negative anti-cyclic citrullinated peptide antibody and rheumatoid factor antibodies, and acute-onset symmetrical polyarthritis with pitting edema. It is often associated with malignancy, which sets it apart from reactive arthritis and seronegative rheumatoid arthritis. Reactive arthritis, in contrast, is a postinfectious asymmetric oligoarthritis commonly triggered by gastrointestinal or genitourinary infections, frequently accompanied by extra-articular manifestations such as conjunctivitis, urethritis, and mucocutaneous lesions. Seronegative rheumatoid arthritis presents with a chronic, progressive, and often erosive symmetrical polyarthritis but lacks the prominent pitting edema seen in RS3PE. In this case, despite the preceding diarrheal illness, the absence of extra-articular symptoms, the presence of symmetrical arthritis with pitting edema, the malignancy association, and the rapid response to steroids strongly support RS3PE over reactive arthritis or seronegative rheumatoid arthritis.

Malignant tumors and RS3PE syndrome frequently coexist; however, cancer is sometimes detected later during the course of RS3PE syndrome.^[9,10]^ This association suggests a paraneoplastic origin. A long-term follow-up study found that cancer developed more frequently in individuals previously diagnosed with RS3PE than in those with sex- and age-matched backgrounds in the same geographic location.^[9]^ It may coexist with, or even precede, a wide range of hematological and solid-organ malignancies.^[4]^ The associated hematological cancers include Hodgkin lymphoma, leukemia, myelodysplastic syndrome, and angioimmunoblastic T-cell leukemia, while related solid tumors include those of the prostate, gastrointestinal tract, lungs, breasts, ovaries, bladders, and endometrium.^[4]^ Interestingly, our patient had 2 different types of malignancy: papillary thyroid carcinoma and adenocarcinoma of unknown origin. The paraneoplastic form of RS3PE should be suspected in any patient, especially if the syndrome is accompanied by systemic symptoms such as fever, weight loss, and anorexia, as in the case of our patient.

Most patients with RS3PE respond to low-to-moderate doses of glucocorticoids. However, patients with underlying malignancies may require higher doses. The mean time to resolution was 133 ± 123 days, with improvement noted within a week of starting treatment in most cases.^[1,4,10]^ Other medications have been used in the management of RS3PE with varying degrees of effectiveness, including hydroxychloroquine, colchicine, and unidentified disease-modifying antirheumatic drugs (DMARDs).^[4]^ Our patient responded well to a moderate dose of prednisolone. However, when the dose was tapered further, the patient relapsed and developed active symptoms. Based on these previous reports, we considered adding DMARDs and selected hydroxychloroquine, which is the most used DMARD, to be the most appropriate option. It is well known that this syndrome usually responds to low-dose corticosteroid treatment and remains in remission for a long period unless accompanied by cancer, in which case it tends to respond poorly to steroid therapy.^[4,9,10]^

In conclusion, patients with RS3PE should be evaluated for simultaneous malignancies to enable early and effective cancer treatment. However, such screening standards are lacking. Therefore, further research is required to develop accessible, reliable, and cost-effective screening procedures for these patients. RS3PE can be accompanied by 2 different types of malignancies, such as concomitant papillary thyroid carcinoma and adenocarcinoma of unknown origin, as in the case of our patient. Patients presenting with RS3PE and significant systemic symptoms or requiring higher doses of corticosteroids should be suspected of having concomitant malignancies. The most important treatment in such cases is surgical resection of the tumor, chemotherapy, or both, as was the case with our patient. Our case highlights the importance of physicians becoming aware of the distinguishing signs and symptoms of RS3PE and investigating any patient suspected of having RS3PE for hidden cancers. Moreover, with regard to treatment, hydroxychloroquine could be a good option as a DMARD for the treatment of paraneoplastic RS3PE. More studies are needed in the future to confirm these findings.

## Acknowledgments

We would like to thank Editage (www.editage.com) for English language editing.

## Author contributions

**Conceptualization:** Samar Alharbi.

**Data curation:** Samar Alharbi.

**Formal analysis:** Samar Alharbi.

**Writing – original draft:** Samar Alharbi.

**Writing – review & editing:** Samar Alharbi.
